# Aluminum phosphide poisoning: Effect of correction of severe metabolic acidosis on patient outcome

**DOI:** 10.4103/0972-5229.53111

**Published:** 2009

**Authors:** S. Jaiswal, R. K. Verma, N. Tewari

**Affiliations:** **From:** Department of Anesthesiology, Institute of Medical Sciences, Banaras Hindu University, Varanasi - 221 005, India

**Keywords:** Aluminum phosphide, arterial blood gases, poisoning, sodium bicarbonate

## Abstract

Forty patients of aluminum phosphide poisoning who were admitted to the ICU of Sir Sunder Lal Hospital, Banaras Hindu University, were studied. Restlessness, excessive thirst, shock, arrhythmias, tachypnoea, and severe metabolic acidosis were the common clinical findings. Only repeated and full correction with intravenous sodium bicarbonate was able to cope up with the severity and rapidity of acidosis. There was no significant change in blood pressure, pulse rate, and respiratory rate after full correction but gradually pulse and systolic blood pressure settled after ionotropic support in the survivors. There was significant improvement from 30.36% in the case when only half correction was done, as has been the common practice, to 57.5%, when full correction of metabolic acidosis was done.

## Introduction

India being an agricultural country has always faced the problem of storage of food grains. Aluminum phosphide, a commonly used grain preservative and fumigant, is available in airtight containers so as to maintain its freshness and activity. Once the container is opened, tablets get exposed and on coming in contact with the moisture, phosphine gas is liberated. In India it is being used as a grain preservative since long, but as a suicidal agent, it has been known since early 80's.[1] This poisoning has now acquired epidemic proportions.[2]

The present study was conducted with the aim to see:

1. The effect of full correction of severe metabolic acidosis in aluminum phosphide poisoning by intravenous sodium bicarbonate. To see its ability to cope up with the rapidity and severity of acidosis.
2. The effect of full correction of acidosis on survival rate in aluminum phosphide poisoning.

## Materials and Methods

The present study was conducted on 40 patients with aluminum phosphide poisoning. The patients were admitted to the emergency OPD, Sir Sunderlal Hospital, Banaras Hindu University. For diagnosis of aluminum phosphide poisoning, history of intake of aluminum phosphide and clinical features, like nausea, vomiting, restlessness, excessive thirst, epigastric pain, hypotension, garlic odor in breath, and tachypnoea, were taken into account. All patients were shifted to ICU without undue delay.

Patients with any pre-existing medical illness, doubtful history, absence of all clinical features of aluminum phosphide poisoning, and features of concomitant poisoning with any other agent were excluded from the study.

History was taken regarding the number of tablets, freshness of tablets, mode and reason of ingestion, nausea, and vomiting. Gastric lavage was done immediately. On arrival of the patient to ICU, the following parameters were monitored – pulse, NIBP, SaO_2_, ABG, respiratory rate, and urine output. Resuscitation of the patient was done on the line of breathing and circulation. Immediate investigation of arterial blood gases, Na^+^, K^+^, and ionized Ca^++^ was done by I-Stat portable blood gas analyzer. Metabolic acidosis was corrected by intravenous sodium bicarbonate, requirement of which was guided by Base Excess.

NaHCO_3_ required (meq) = 0.6 × body weight (kg) × Base Excess

Total requirement of sodium bicarbonate was given immediately (full correction) and arterial blood gases were estimated after one hour and sodium bicarbonate was again given accordingly. After that, arterial blood gas analysis was done again at one-hour intervals till two consecutive readings of arterial blood pH above 7.4 were obtained. Following which, arterial blood gas analysis was done at 2-, 4-, or 6-hourly intervals, according to requirement. Associated electrolyte imbalances were treated accordingly.

All the patients were given injection hydrocortisone 200 mg IV on arrival and then 150 mg at 8-hourly intervals. Ionotropic support was given with injections dopamine and dobutamine.

Postmortem examination was conducted in all expired patients. Tissue samples were taken from heart, lung, liver, and kidney and were sent for histopathological examination. The data were statistically analyzed.

## Results

Forty patients were included in the study, of whom 22 survived and 18 expired. The survival rate was 55%.

### Demographic data

The mean age in the study group was 24 ± 4.89 years.There was no significant difference in age among survivors and nonsurvivors. Males were more common in the study group and more male patients survived [Table 1].

**Table 1:** Demographic data

	Total (N = 40) Mean ± SD	Survivors (n = 22) Mean ± SD	Nonsurvivors (n = 18) Mean ± SD
Age in years	24.00 ± 4.89	24.14 ± 6.94	23.79 ± 3.53
Sex ratio (M:F)	26:14	16:6	10:8

In our study, 33 patients took fresh tablets and 7 patients took exposed tables. The survival rate was 45.5% in patients who had fresh tablets while it was 100% in those who consumed exposed tablets. Average number of fresh tablets among survivors was 1.14 ± 0.38 whereas it was 2.33 ± 1.32 among nonsurvivors. The difference was statistically significant [Table 2].

**Table 2:** Number of tablets consumed

Fresh tablets			Exposed tablets		

Total Mean ± SD *n* = 33	Survivors Mean ± SD n = 15	Nonsurvivors Mean ± SD n = 18	Total Mean ± SD n = 7	Survivors Mean ± SD n = 7	Nonsurvivors Mean ± SD n = 0
1.81 ± 1.17	1.14 ± 0.38	2.33 ± 1.32	1.74 ± 1.50	1.74 ± 1.50	

### Heart rate

There was wide variation in pulse rate among both survivors and nonsurvivors. Average pulse rate on arrival was 100.0 ± 22.23/min (range, 62–142). There was no significant difference of pulse rate between survivors and nonsurvivors on arrival. Significant difference was found at 6–12 hour and 24–48 hours duration [Table 3]

**Table 3:** Heart rate per minute

Time (hours)	Range (beats/min)	Total Mean ± SD, (*n*)	Survivors Mean ± SD, (*n*)	Nonsurvivors Mean ± SD, (*n*)	t	P
0	62–142	100.0 ± 22.23 (40)	96.3 ± 26.5 (n = 22)	102.1 ± 16.94 (18)	0.38	>0.05
1–6	68–136	101.4 ± 20.10 (36)	97.2 ± 23.93 (22)	108.0 ± 10.30 (14)	1.12	>0.05
6–12	68–136	100.8 ± 23.51 (26)	95.1 ± 20.59 (22)	132.5 ± 4.95 (4)	2.48	>0.05
12–24	65–122	94.9 ± 20.98 (24)	92.8 ± 20.64 (22)	118.0 ± 0.0 (2)	1.17	>0.05
24–48	62–118	86.5 ± 17.24 (24)	83.6 ± 14.79 (22)	118.0 ± 0.0 (2)	2.23	<0.05

### Mean arterial pressure

The average mean arterial pressure on arrival was 73.1 ± 11.52 mm Hg. It was significantly high among survivors (73.1 ± 11.52 mm Hg) as compared to nonsurvivors [Table 4].

**Table 4:** Mean arterial pressure

Time (hours)	Range (mm Hg)	Total Mean ± SD, (*n*)	Survivors Mean ± SD, (*n*)	Nonsurvivors Mean ± SD, (*n*)	t	P
0	60–91	73.1 ± 11.52 (18)	73.1 ± 11.52 (18)	− (0)	-	-
1–6	62–90	75.4 ± 10.12 (10)	75.4 ± 10.12 (20)	− (0)	-	-
6–12	63–88	75.4 ± 8.88 (24)	75.4 ± 8.36 (22)	62.0 ± 0.0 (2)	1.55	>0.05
12–24	70–92	79.5 ± 7.58 (22)	79.5 ± 7.58 (22)	− (0)	-	-
24–48	73–100	83.7 ± 7.11 (22)	83.7 ± 7.11 (22)	− (0)	-	-

### Respiratory rate

Mean respiratory rate at arrival was 25.7 ± 9.64/min. There was no significant difference in respiratory rate among survivors and nonsurvivors. The difference became significant during 12–24 hours and 24–48 hours duration [Table 5].

**Table 5:** Respiratory rate

Time (Hours)	Range (breaths/min)	Total Mean ± SD, (*n*)	Survivors Mean ± SD, (*n*)	Nonsurvivors Mean ± SD, (*n*)	t	P
0	12–42	25.7 ± 9.64 (40)	24.3 ± 9.31 (22)	27.4 ± 10.31 (18)	0.71	>0.05
1–6	16–39	25.8 ± 5.67 (36)	24.9 ± 4.64 (22)	27.4 ± 7.11 (14)	0.91	>0.05
6–12	17–35	24.1 ± 5.46 (26)	23.4 ± 4.79 (22)	28.0 ± 7.01 (4)	1.19	>0.05
12–24	16–34	23.2 ± 5.38 (24)	22.3 ± 4.38 (22)	34.0 ± 0.00 (2)	2.56	<0.05
24–48	15–32	20.4 ± 5.41 (22)	19.3 ± 3.73 (22)	32.0 ± 0.00 (2)	3.26	<0.01

### Acid-base study

All patients in this study showed severe metabolic acidosis. Base Excess on arrival was −15.7 ± 3.34 (range, −21 to −11). There was no significant difference in Base Excess among survivors (−14.6 ± 3.14) and nonsurvivors (−17.1 ± 3.22). Metabolic acidosis was adequately corrected during stay in ICU. The difference became significant during 12–24 hours and 24–48 hours [Table 6].

**Table 6:** Base Excess

Time (Hours)	Range	Total Mean ± SD, (n)	Survivors Mean ± SD, (n)	Nonsurvivors Mean ± SD, (n)	t	P
0	−21 to −11	−15.7 ± 3.34 (40)	−14.6 ± 3.14 (22)	−17.1 ± 3.22 (18)	1.75	>0.05
1–6	−10 to +7	0.12 ± 4.26 (34)	0.8 ± 4.74 (22)	−1.3 ± 3.08 (12)	0.97	>0.05
6–12	−10 to +3	−0.9 ± 3.33 (26)	−0.72 ± 3.35 (22)	−2.0 ± 3.0 (4)	0.52	>0.05
12–24	−7 to +1	−0.8 ± 2.59 (24)	−0.3 ± 1.79 (22)	−7.0 ± 0.0 (2)	3.58	<0.01
24–48	−8 to +3	−0.7 ± 2.74 (24)	0.0 ± 1.55 (22)	−8.0 ± 0.0 (2)	4.91	<0.01

### pH

Average pH was 7.201 ± 0.143 on arrival. There was no significant difference in pH among survivors and nonsurvivors. The difference became significant at 12–24 hours and 24–48 hours [Table 7]. There was significant difference in requirement NaHCO_3_ between survivors (554 ± 128.33° ml) and nonsurvivors (722.22 ± 253.85° ml). Most of the patients (65%) required 400–600° ml of NaHCO_3_ [Table 8].

**Table 7:** pH

Time (hours)	Range	Total Mean ± SD (*n*)	Survivors Mean ± SD, (*n*)	Nonsurvivors Mean ± SD, (*n*)	t	P
0	6.605–7.390	7.201 ± 9.64 (40)	7.250 ± 0.083 (22)	7.142 ± 0.181 (18)	1.77	>0.05
1–6	6.830–7.554	7.424 ± 0.146 (36)	7.480 ± 0.039 (22)	7.321 ± 0.213 (14)	2.48	<0.05
6–12	7.392–7.505	7.440 ± 0.048 (26)	7.449 ± 0.047 (22)	7.393 ± 0.00 (4)	1.65	>0.05
12–24	7.289–7.451	7.417 ± 0.043 (24)	7.430 ± 0.015 (22)	7.289 ± 0.00 (2)	8.81	<0.001
24–48	7.269–5.453	7.408 ± 0.045 (24)	7.421 ± 0.012 (22)	7.269 ± 0.00 (2)	11.69	<0.001

**Table 8:** NaHCO_3_ requirement

Volume (ml)	Total (*N* = 40)	Survivors (*n* = 22)	Nonsurvivors (*n* = 18)
Mean ± SD	630 ± 207.99	554.6 ± 129.33	722.22 ± 253.85

## Discussion

There are only a few case reports and studies on aluminum phosphide poisoning. Most of these studies are related to investigation findings and only a few studies are concerned with therapeutics. Till now, there is no reported study concentrating on correction of metabolic acidosis in this poisoning.

The incidence of aluminum phosphide poisoning is increasing day by day. Number of patients admitted to our ICU with this poisoning have been on the rise, in 2003 – 11 patients, in 2004 – 10 patients, in 2005 – 23 patients, in 2006 – 25 patients, and in 2007 – 42 patients with survival rates of 27.27, 10, 34.78, 32, and 30.95%, respectively. Overall, 112 patients were admitted in our ICU during these five years with the average survival rate of 30.36%. These patients were treated with half correction of metabolic acidosis. In the present study full correction of metabolic acidosis was done on arrival and during stay in ICU. Out of 40 patients, 22 survived showing an improvement in survival rate, i.e. 55%.

Most of the patients in our study were in their twenties (70%). In the same fashion Misra *et al,*[3] reported that mean age of patients in their study was 23 years. Chugh *et al,*[4] and Singh *et al,*[5] have also reported that aluminum phosphide poisoning is more common in younger generation.

Most of the patients in this study were males (70%). There was no significant difference in age and sex among patients who survived or expired. Thirty two patients (80%) in the present study had taken fresh tablets. This shows easy availability of fresh aluminum phosphide tablets pack due to unrestricted sale. Eight patients (20%) had taken exposed tablets and all of them survived. Thus, freshness and number of tablets are the most important features influencing toxicity and outcome.

Shock has been found to be the commonest and most important clinical feature in phosphine poisoning. Thirty five percent (14 out of 40) of patients had unprocurable systolic blood pressure at presentation. Out of 14, only two patients could be saved. With treatment there was a gradual increase in blood pressure. Most of the patients had blood pressure refractory to ionotropic support. Shock has been described by many authors as the commonest manifestation and lead cause of death. This is thought to cause myocarditis.[6,7] There was no significant change in blood pressure immediately after correction of acidosis.

In the present study there has been a wide variation in pulse rate (range, 62–142). Patients had shown bradycardia as well as tachycardia. This finding coincides with the findings of Chugh *et al.* [4] There was no significant difference in pulse rate among survivors and nonsurvivors. Pulse rate settled gradually among survivors. There was no significant immediate change in pulse rate after correction of acidosis.

All the patients in the present study have shown an increase in respiratory rate. One patient was gasping at the time of arrival. He was immediately intubated and IPPV was started. The increase in respiratory rate was due to metabolic acidosis (compensatory response). There was no significant immediate change in respiratory rate after correction of acidosis. There was significant difference in respiratory rate between survivors and nonsurvivors at 24–48 hours.

In this study, there was severe metabolic acidosis in all the patients (Base Excess range −21 to −11). It was adequately controlled by full correction of acidosis by NaHCO_3_. pH and Base Excess have remained near-normal. Average total volume of NaHCO_3_ given to the patient was 630 ml. In 30% of patients there was ECG abnormality at arrival. All these patients survived. Commonest ECG findings were ST segment depression and T wave inversion. These changes were reversed with correction of acidosis in 70% of patients.

## Conclusion

On the basis of above study we can conclude that more structured trials are needed in this poisoning because since even with this improved outcome figure in our study mortality rate is still very high. It is also very important to recognize that even with our ‘Aggressive Correction of Acidosis’ protocol, the predictability of outcome is very poor. Strict legislations are needed to control sale of aluminum phosphide over the counter. It should be sold in better designed packs which can liberate phosphine slowly while it should be difficult to ingest it.
